# Cumulative Live-Birth Rates by Maternal Age after One or Multiple *In Vitro* Fertilization Cycles: An Institutional Experience

**DOI:** 10.22074/ijfs.2020.5855

**Published:** 2020-02-25

**Authors:** Dalia Khalife, Anwar Nassar, Ali Khalil, Johnny Awwad, Antoine Abu Musa, Antoine Hannoun, Lina El Taha, Fatin Khalifeh, May Abiad, Ghina Ghazeeri

**Affiliations:** 1 Department of Obstetrics and Gynecology, American University of Beirut Medical Center, Beirut, Lebanon; 2 Faculty of Medicine, American University of Beirut, Beirut, Lebanon

**Keywords:** Assisted Reproductive Techniques, Live Birth Pregnancy Rate, Maternal Age, Multiple Pregnancy

## Abstract

**Background:**

The aim of this retrospective study is to investigate the cumulative live birth rate (CLBR) following one or more completed *in vitro* fertilization (IVF) cycles (up to 6 cycles) stratified by maternal age and type of infertility.

**Materials and Methods:**

In this retrospective study, five hundred forty-seven women who received 736 fresh ovarian stimulation/embryo transfer cycles between January 2016 and December 2016 were included in the study at a tertiary care center located in Lebanon.

**Results:**

In all women, the live birth rate for the first cycle was 33.0% [95% confidence interval (CI): 27.8-38.2]. The CLBR showed an increase with each successive fresh cycle to reach 56.9% (95% CI: 51.2-62.4) after 3 cycles and 67.9% (95% CI: of 62.5-73.0) after 6 cycles. The CLBR following 6 cycles reached 69.9% (95% CI: 63.8-75.6) in women younger than 35 years. In women older than 40 years, however, the live birth rate for the first cycle was signifi- cantly low at 3.1% (95% CI: 0.3-9.5) with a plateau in success rates after 4 cycles reaching 21.9% (95% CI: 9.2-40.0). Couples with different types of infertility had CLBRs ranging from 65% to 72%, with the exception of women with low ovarian reserve, where CLBRs reached 29.4% (95% CI: 10.3-56.0).

**Conclusion:**

The CLBR at a referral center in a Middle Eastern country reached 67.9 % after 6 cycles, with variations by age and type of infertility treatment. These findings are encouraging for patients insisting to extend their treatment beyond 4 to 5 cycles.

## Introduction

The prevalence of infertility is around 9% worldwide (1), while it is 10-15% in the Middle East (ME) for many reasons, including a high incidence of postpartum infections, iatrogenic tubal and pelvic infertility and women delaying childbearing (2, 3). The number of women treated with *in vitro* fertilization (IVF) in the ME has increased from 8305 cycles in 2005 to 11876 cycles in 2008 (4). The live birth rate per cycle is the ultimate success, and therefore it has been used in multiple studies (5-7). The outcome as livebirth per fresh IVF cycle is more evocative for patients coming for counseling, than the outcome as a positive pregnancy test per cycle. However, the best way is to counsel patients about the cumulative chances of success after a defined number of IVF cycles (8). Some centers who have adopted the single embryo transfer policy have reported cumulative live birth rates (CLBRs) as a fresh embryo transfer cycle followed by cryo-warmed cycles, all resulting from one episode of ovarian stimulation (8-12). On the other hand, others have included only fresh cycles for CLBR assessment (6, 13-15). Although it has been previously reported that the live birth rates decrease after the 4th cycle (13, 16), there is no medical reason behind limiting the number of cycles. Many patients are likely to discontinue their infertility treatments because of the psychological burden of the process and the cost of repetitive failed IVF cycles (17). On the other hand, the decision of the couple to proceed with further fresh cycles is bounded by cultural factors where the continuation of marriage is dependent on having children and many couples are reluctant to seek egg or sperm donation cycles for ethical and religious reasons.

To the best of our knowledge, CLBR after IVF/intra-cytoplasmic sperm injection (ICSI) cycles has never been reported at a national level in Lebanon, nor in the ME. It is important to determine these rates and how they change with repeated cycles, according to maternal age and type of infertility. It is essential to define an IVF cycle for these patients as the initiation of ovarian stimulation with subsequent fresh embryo transfer.

We aim to determine whether the CLBR increases over multiple successive IVF cycles, providing patients with a better estimation of their chances of a live birth.

## Materials and Methods

### Ethical approval

The Ethical approval for this study was obtained from the Institutional Review Board at the AUBMC (BIO – 2017- 0331).

### Study population

This retrospective cohort study was performed on all patients scheduled to have fresh IVF/ICSI cycles at the AUBMC between January 2016 and December 2016. One IVF cycle is defined as a fresh embryo transfer attempt resulting from one episode of ovarian stimulation. All embryo transfers involving the transfer of one or more embryos were included in the study to reproduce the daily practice of assisted reproductive technologies in our region.

Cycles that were excluded are those which were cancelled before the oocyte retrieval or before the embryo transfer, patients who had their IVF cycles after December 2016 and cycles with frozen embryos/frozen oocytes. Cancellation rate was 5%.

### Baseline characteristics

Baseline characteristics included different age categories (≤35, 36-39 and ≥40 years) and different types of infertility (male factor, unexplained infertility, ovulatory disorders, endometriosis, low ovarian reserve, tubal infertility and combined factors). Data collected included levels of anti-müllerian hormone and/or day 3 follicle stimulating hormone (FSH) and estradiol.

### Fresh embryo transfer

Patients underwent controlled ovarian stimulation and oocyte retrieval after 10-12 days of stimulation. All cycles included were ICSI cycles. Fresh embryo transfer took place two, three or five days after the oocyte retrieval. All cycles with pre-implantation genetic testing (PGT) or frozen embryo transfer were excluded.

### Outcomes

Live birth and CLBRs per cycle were the main outcome measures, stratified by maternal age and type of infertility in up to six IVF cycles. Live birth was defined as a newborn delivered after 24 weeks of gestation. Once a woman succeeded in achieving her first live born baby from IVF, she does not contribute further to the cumulative rates calculation. All women without a live birth in a previous cycle were eligible for a subsequent cycle. The CLBR at one cycle expressed the likelihood of a live birth at that cycle and from all preceding cycles.

### Statistical analysis

For all patients included, descriptive statistics of demographics and treatment characteristics were analyzed. A summary of the statistics was prepared as percentages for categorical variables and is compared using the chi-square test. The mean ± standard deviation (SD) was used for continuous variables and was compared using Student's t test or one-way analysis of variance (ANOVA).

The primary outcome of this study was the CLBR. Patients were not re-enrolled after having a first live birth in a previous IVF cycles.

The live birth rate per fresh IVF treatment was calculated at different number of cycles, through dividing the number of women in each cycle who had their first live birth by the total number of IVF cycles. Conservative CLBR was also calculated by dividing the total number of women who had their first live birth up to the corresponding cycle by the total number of women who ever attempted IVF (18). The binomial distribution was used to calculate the 95% confidence intervals. A log-rank test compared the live birth rate and CLBR within each cycle and across all cycles.

Statistical analysis and computations were performed using Statistical Package for Social Sciences (SPSS IBM version 24 software, AUBMC, Lebanon), and a value of P<0.05 was considered to be statistically significant.

## Results

**Fig 1:**
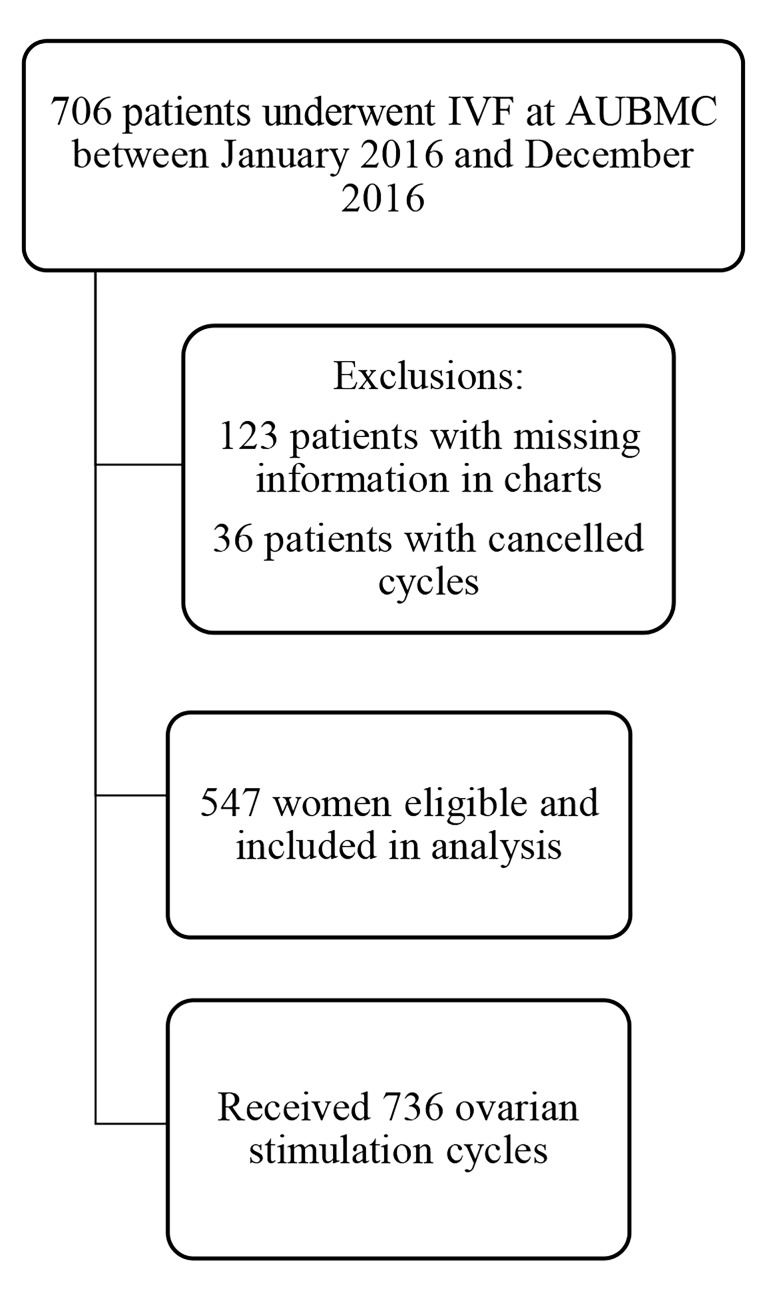
Flow chart of eligible cycles. AUBMC; American University of Beirut Medical Center.

In this cohort study a total of 706 women underwent fresh IVF cycles at the AUBMC from January 2016 to December 2016. After exclusions, 547 women with 736 fresh ovarian stimulation cycles were included in the analysis (Fig .1), with a yield of 10.4 ± 7.8 oocytes retrieved per cycle.

Tables 1 and 2 summarize the baseline characteristics of the cohort. Sixty-five percent of the patients undergoing IVF cycles were younger than 35 years of age. The mean duration of infertility was 4.2 years with male infertility being the most frequent diagnosis (42.5%).

**Table 1:** Characteristics of the 736 fresh IVF Cycles at the American University of Beirut Medical Center in 2016


Variables	For all cycles number (%)

Nationality	
Lebanese	669 (91.1)
Syrian	32 (4.4)
Iraqi	20 (2.7)
Others	13 (1.7)
Age (Y)	
≤35	479 (65.1)
36-39	137 (18.6)
≥40	120 (16.3)
Medical history	
Healthy	629 (86.0)
Thyroid disorder	47 (6.4)
Smoking status	
Non-smoker	587 (81.3)
Menstrual regularity	
Regular	652 (88.6)
Type of infertility	
Primary	425 (58.2)
Secondary	308 (41.8)
Cause of infertility	
Male factor	311 (42.5)
Unexplained infertility	136 (18.6)
Combined factors	97 (13.3)
Ovulatory disorder	55 (7.5)
Endometriosis	51 (7.0)
Low ovarian reserve	48 (6.6)
Tubal factor	33 (4.5)
Total number of cycles	
1	318 (43.2)
2	172 (23.4)
3	102 (13.9)
4	64 (8.7)
5	39 (5.3)
6	41 (5.6)
COS	
Antagonist protocol	631 (85.7)
Long protocol	85 (11.5)
Mild stimulation	19 (2.7)
Trigger	
hCG trigger	574 (78.0)
GnRHa trigger	159 (22.0)
Day of embryo transfer	
Day 2	139 (19.0)
Day 3	454 (62.0)
Day 5	139 (19.0)


Spring (March to May), Summer (June to August), Fall (September to November), Winter (December to February). IVF; In vitro fertilization, COS; Controlled ovarian stimulation, hCG; Human chorionic gonadotropin, and GnRHa; Gonadotropin releasing hormone agonist.

**Table 2:** Characteristics of the 736 fresh IVF Cycles at the American University of Beirut Medical Center in 2016


Variables	For all cycles

BMI (Kg/m^2^)	25.5 ± 4.7
Duration of infertility (Y)	4.2 ± 3
Day 3 FSH (mIU‎/mL)	7.1 ± 2.6
Day 3 Estradiol (ng/mL)	63.5 ± 62.0
AMH (ng/mL)	2.1 ± 2.3
Number of oocytes retrieved	10.4 ± 7.8
Number of mature oocytes	7.4 ± 5.2
Number of 2PN zygotes on day 1	5.5 ± 3.9
Number of embryos transferred	2.7 ± 0.9


Data are presented as mean ± SD. IVF; In vitro fertilization, BMI; Body mass index, FSH; Follicle-stimulating hormone, AMH; Anti-mullerian hormone, and PN; Pronuclear.

Cycles were stimulated with various protocols, with the antagonist protocol being the most commonly used (85.7%). Final oocyte maturation was mainly triggered by human chorionic gonadotropin (hCG) (78% of cycles), while the remaining cycles were triggered by gonadotropin releasing hormone agonist (GnRH) agonist. Transvaginal oocyte collection was performed 35-36 hours after the trigger. The luteal phase was supported by vaginal (micronized progesterone suppositories), intra-muscular and/or oral progesterone (Dydrogesterone).

The average number of embryos transferred per patient was 2.7, and 81% of the embryo transfers were performed on day 2 or day 3 with a fresh cleavage-stage embryo. This resulted in 216 live births (29.3%), where 61.6% were singletons and 38.4% were multiple gestations (Table S1, See Supplementary Online Information at www.ijfs.ir).

### Cumulative live birth rates

The overall CLBR for all treatment cycles and all age groups is shown in Figure S1 (See Supplementary Online Information at www.ijfs.ir). The conservative CLBRs across all cycles up to cycle number 6 were calculated (Table 3). Overall, the live birth rate resulting from the first fresh IVF cycle is 33.0% (95% CI: 27.8-38.2). This value remained above 20% up to the sixth cycle. The conservative CLBR showed an increase with each successive fresh cycle to reach 56.9% (95% CI: 51.3-62.4) after 3 cycles and 67.9% (95% CI: 62.5-73.0) after 6 cycles.

Conservative CLBR stratified for the different age groups are presented in Figure S2 (See Supplementary Online Information at www.ijfs.ir) and in Table 4. The live birth rates fluctuated with an overall decrease with progressive cycles and in patients younger than 35 years were 37.4%, 34.2%, 30.6%, 34.5%, and 33.3% at cycles 1 through 5, respectively. Following 6 cycles, CLBRs reached 69.9% (95% CI: 63.8-75.6) in patients younger than 35 years and 83.7% (95% CI: 69.3-93.2) in patients between 36 and 39 years old. The CLBR decreased after the age of 40, as a plateau in success rates was reached after the 4^th^ cycle with 21.9% (95% CI: 9.3-40.0). The log-rank test revealed significantly different age-specific rates (P<0.05).

**Table 3:** Live birth rates within initiated treatment cycle and conservative cumulative live birth rates across all cycles


Cycle number	Number of cycles	Number of live births	Live birth rate with-in each cycle, % (95% CI)	Cumulative live birth rates across all cycles, % (95% CI)

1	318	105	33.0 (27.8-38.2)	33.0 (27.8-38.2)
2	172	49	28.5 (21.7-35.3)	48.4 (42.8-54.1)
3	102	27	26.5 (17.8-35.2)	56.9 (51.3-62.4)
4	64	16	25 (14.1-35.9)	61.9 (56.4-67.3)
5	39	8	20.5 (7.2-33.8)	64.5 (58.9-69.7)
6	41	11	26.8 (12.7-41.0)	67.9 (62.5-73.0)


CI; Confidence interval

**Table 4:** CLBRs across all age groups


Cycle number	Number of cycles	Number of live births	Live birth rate within each cycle, % (95% CI)	Cumulative live birth rates across all cy-cles, % (95% CI)

1. Women aged ≤35 years’ old				
1	243	91	37.4 (31.3-43.6)	37.4 (31.3-43.6)
2	117	40	34.2 (25.5-42.9)	53.9 (47.4-60.3)
3	62	19	30.6 (18.8-42.4)	61.7 (55.3-67.9)
4	29	10	34.5 (16.1-52.9)	65.8 (59.5-71.8)
5	18	6	33.3 (9.2-57.5)	68.3 (62.1-74.1)
6	10	4	40.0 (3.1-76.9)	69.9 (63.8-75.6)
2. Women aged 36-39 years’ old				
1	43	13	30.2 (15.9-44.5)	30.2 (15.9-44.5)
2	32	9	28.1 (11.7-44.6)	51.2 (35.5-66.7)
3	22	4	18.2 (0.7-35.7)	60.5 (44.4-75.0)
4	20	4	20.0 (0.8-39.2)	69.8 (53.9-82.8)
5	11	2	18.2 (0.9-45.4)	74.4 (58.8-86.5)
6	9	4	44.4 (3.9-85.00)	83.7 (69.3-93.2)
3. Women aged ≥ 40 years’ old				
1	32	1	3.1 (0.3-9.5)	3.1 (0.3-9.5)
2	23	0	0	3.1 (0.3-9.5)
3	18	4	22.2 (0.9-43.5)	15.6 (5.3-32.8)
4	15	2	13.3 (-6.1-32.8)	21.9 (9.3-40.00)
5	10	0	0	21.9 (9.3-40.00)
6	22	3	13.6 (-1.9-29.2)	31.2 (16.1-50.0)


CLBRs; Cumulative live birth rate and CI; Confidence interval.

Conservative CLBR categorized by the different types of infertility are presented in Figure S3 (See Supplementary Online Information at www.ijfs.ir). With the exception of women with low ovarian reserves, couples with different types of infertility have a similar live birth rate at the first cycle when compared to all other cycles. The CLBR after 6 cycles for couples with low ovarian reserves is the lowest with 29.4% (95% CI: 10.3-56.0).

## Discussion

This 1-year cohort showed significant CLBRs based on fresh IVF cycles, even in women older than 40 years of age. These numbers can help physicians counsel patients about the chances of successful live births in terms of age and type of infertility with repeated cycles. Because of the health system differences between the ME and Western countries (financial constraints, lack of insurance coverage, ethical and religious reasons), we assessed the CLBRs in fresh IVF cycles only. We chose 6 cycles, because of the significant reduction in success in CLBRs after 4 to 6 cycles noted in the literature (6, 13). Moreover, the number of patients receiving more than 6 cycles is low. In this study, the CLBRs following 1 to 6 successive IVF cycles in a referral tertiary center in the ME was calculated. The conservative estimates of the CLBR increased by more than 50% from cycle number 1 (33.0%, 95% CI: 27.8-38.2) to cycle number 6 (67.9%, 95% CI: 62.5-73.0) across all cycles, whilst it increased by 53.5% in patients who were ≤35 years old, by 36% in patients between 36 and 39 years of age and by only 10% in patients ≥40.

It is believed that the success rate within a cycle decreases with an increase in the number of cycles (5), however, the cumulative rates in our cohort increased up to the sixth cycle. The cumulative rates also increased up to the fourth cycle in women aged ≥40 years old (21.9%, 95% CI: 9.3-40.00). Occasional live births were achieved in patients older than 40 with a probability of 3.1% per started cycle in our cohort compared to 0.46% in a singlecenter Japanese cohort study (19). These findings are in line with a study published by Smith et al. (20) who categorized women older than 40 years of age into 2 groups and showed that women aged 40 to 42 still have acceptable chances up to the ninth cycle, while women older than 42 show an increase up to the fifth cycle only. The same authors also showed that patients with a low yield of oocytes retrieved in previous cycles still benefit from continuing successive cycles if they are younger than 40 years. On the contrary, we showed that when including all reproductive ages in the study, patients with low ovarian reserve and low number of oocytes retrieved have the lowest cumulative rates, plateauing after the second cycle with a 29.41% chance of success. Moreover, our rates were similar to those reported in previous studies, as the CLBRs decreased in older ages (21).

When the cause of infertility was taken into account, the differences noted in CLBRs were insignificant among patients with male factor, unexplained, tubal and combined infertility. In addition, couples with a male factor had the highest CLBRs as it is also outlined in the biggest US study by Luke et al. (22). Furthermore, it is worth mentioning that in patients with anovulation the CLBRs reach plateaus after the third cycles at 65.5%. These results may be explained by the distorted steroidogenesis of the theca cells and metabolic imbalance found in patients with polycystic ovary syndrome (PCOS). The quality of the oocytes has previously been showed to be poorer in patients suffering from PCOS and the finest dosage of ovarian hormonal stimulation in patients undergoing IVF is still debatable (23). Thus, multiple new therapies are implemented in order to improve pregnancy outcomes in this subcategory of patients. Among them, myo-inositol has a pivotal role in cellular signaling, as it has been shown to improve glucose uptake and FSH signaling affecting positively the oocyte quality (24). Nonetheless, data is not strong enough to support this improvement in pregnancy outcomes and additional clinical trials are needed in this regard (24-26).

Only patients with low ovarian reserve had their CLBR plateauing after the second cycle with only 29.4%, which is significantly different from the rest of our study cases mentioned here. With an improvement in cumulative rates of only 7% after 2 cycles and subsequent stabilizing after 6 consecutive cycles, it may be concluded that assisted reproductive technologies in patients with low ovarian reserves may be futile and especially after 3 cycles. Nevertheless, the number of events in this particular group was too small to draw definite conclusions. These findings contradict previous reports that showed no substantial differences in the CLBRs among women with various causes of infertility (27-30).

These results show that for patients willing to continue their treatment, the CLBRs after 6 cycles would be 69.9% (95% CI: 63.8-75.6) at the age of 35 years or younger, which is close to the live birth rate of 75% in a woman trying to conceive naturally. However, the CLBR at the age of 40 years for our subjects is 31.2% (95% CI: 16.1- 50.0), which is slightly lower than the 44% of natural conception (31, 32). Considering the age-related reduction in success rates in IVF treatments, our results are reassuring that a CLBR up to 83.7% in women aged 36 to 39 years (95% CI: 69.3-93.2) is achievable, encouraging women younger than 40 years to repeat their IVF treatment cycles when the cost is not a barrier to the treatment. Our findings are in line with a previous report showing that patients older than 40 years are less likely to conceive with repeated cycles compared to the younger ones (27), thus patients older than 40 years of age should be adequately counseled that IVF at this point does not improve the agerelated decrease in fertility.

In a retrospective study on 4810 transfers, the possible beneficial effects of transvaginal ultrasound-guided ET was assessed and it was shown that the number of pregnancies per ET significantly increased when performed under transvaginal ultrasound compared to trans-abdominal (38% vs. 30%, P<0.001). Transvaginal ultrasound may simplify difficult transfers via a better monitoring of the trans-cervical area improving the overall technique (33).

The multiple pregnancy rate was 38.4 %, with 83.1% twins, and 15.7% triplets, reflecting the continuing practice of transferring more than 2 embryos in the ME. The mean number of embryos transferred in this study was 2.7 (± 0.9). These rates are high when compared to averages reported in the American and European registries, with only 25.1% risk of multiple births (29). The percentage of multiples is slightly lower than the ones observed in Argentina (43.1%), Brazil (55.9%) and Taiwan (40.5%) (34). This indicates the utmost priority for establishing new policies and regulations regarding the number of embryos transferred per cycle to lower the increased risk of perinatal and maternal morbidity and mortality associated with multiple pregnancies (35). With improvements in cryopreservation methods, consecutive fresh and frozen single-embryo transfer cycles should be encouraged, thus taking into account frozen cycles when estimating CLBRs.

This is the first study in the ME to report CLBRs per cycle following fresh IVF treatment over a one-year period. We classified our patients according to age and the type of infertility when to our knowledge other studies have failed to do so. In addition, we included all patients presenting for their first cycle and undergoing fresh cycles, thus increasing the generalizability of our results. CLBRs were calculated on the basis of conservative estimates reflecting that women who do not achieve a live birth at their first attempt, will have their chances increased after successive attempts. In our study, we used live birth rates as a primary outcome while other studies reported pregnancy rates only (14, 15).

Because of the retrospective aspect of the study, confounders were not reliably controlled, and significant biases affected the outcome. Our study has several other drawbacks. For instance, the cycles that were cancelled before oocyte retrieval were not recorded. This might have led to a minor overestimation of the CLBRs, as patients with severely poor prognosis did not account for the number of cycles and were excluded. However, only 36 patients were deemed ineligible, concluding that our findings are very close to the actual rates and the methodological bias had a relatively small influence on the final results. Patients who usually discontinue treatment are patients with very poor prognosis and are older than 40 years. In our cohort, only 16.3% of the cases were older than 40 years and most women had a high oocyte yield (10.4 ± 7.8). Because of these two important factors, we expect a very small difference between the rates that we calculated and the actual rates. On the other hand, some patients had undergone previous IVF cycles in other centers, adding some bias to the results since different laboratories and techniques may have been used. Furthermore, there was extensive heterogeneity in the different controlled ovarian stimulation protocols used limiting the generalizability of the results.

Our observed results postulate the chances of obtaining a live birth after one or multiple consecutive cycles, basing our decisions on some realistic expectations of CLBRs. In addition, it provides hope for older patients whose CLBRs are not affected by their age up till the age of 40. This reveals the advancements in reproductive technologies with the growth of ICSI (35).

In a region that is highly influenced and controlled by religious beliefs, different barriers exist for using assisted reproductive technologies, preventing the performance of oocyte and sperm donation. Therefore, with these unanticipated findings, couples have no other options except to extend their treatment cycles beyond 4 cycles.

## Conclusion

This study provides an approach for estimating the effectiveness of IVF over 6 successive cycles. We showed an increase in the CLBRs over multiple cycles reaching a 67.9% chance of conception after 6 cycles, with variations by age and type of diagnosis. These findings are reassuring for patients insisting to continue with their treatments given the meaningful cumulative chances of success. Thus, barriers to continuation of treatment should be reduced with improvement in couples’ counseling. Moreover, our results show that IVF treatments approach the natural fertility rates in patients younger than the age of 40.

However, the multiple pregnancy rate is still high in this part of the world due to the lack of regulations and policies. The practice is surrounded by an inequity in accessibility to this expensive form of health resource with fluctuation in the proportion of treatment cycles where few patients have the privilege of starting another IVF treatment in the case of a previous failed one.

## Supplementary PDF


