# Supplementary material for: Cumulative Live-Birth Rates by Maternal Age after One or Multiple In Vitro Fertilization Cycles: An Institutional Experience
Source: Int J Fertil Steril. 2020 Feb 25;14(1):34–40. doi: 10.22074/ijfs.2020.5855 (PMC7139227; doi:10.22074/ijfs.2020.5855)
Supplement: Supplementary file 1 [file Int-J-Fertil-Steril-14-34-s01.pdf]

Int J Fertil Steril
International Journal of Fertility & Sterility
ISSN:2008-076X eISSN:2008-0778

Supplementary Information for

Cumulative Live-Birth Rates by Maternal Age after One or Multiple *In Vitro* Fertilization Cycles: An Institutional Experience

Dalia Khalife, M.D.[1], Anwar Nassar, M.D.[1], Ali Khalil, M.D.[1], Johnny Awwad, M.D.[1], Antoine Abu Musa, M.D.[1], Antoine Hannoun, M.D.[1], Lina El Taha, M.D.[1], Fatin Khalifeh, M.D.[1], May Abiad, M.D.[2], Ghina Ghazeeri, M.D.[1*]

1. Department of Obstetrics and Gynecology, American University of Beirut Medical Center, Beirut, Lebanon
2. Faculty of Medicine, American University of Beirut, Beirut, Lebanon

Table S1: Outcomes of cycles

Variables		For all cycles
Delivery	AUBMC	104 (45.2)
Miscarriage rate		76 (10.3)
Live birth rate		216 (29.3)
Implantation rate		20.9 ± 31.2
Weeks of miscarriage		8.6 ± 4.3
Weeks of delivery		36.7 ± 2.9
Weight of baby at delivery		2799.4 ± 1186.5
Pregnancy complications	No complications	67 (43.8)
	Preterm labor	24 (15.7)
	Gestational diabetes	7 (4.6)
	Hypertension in pregnancy	5 (3.3)
	Pre-eclampsia	8 (5.2)
	Placental abruption	2 (1.3)
	First trimester bleeding	13 (8.5)
	Second trimester bleeding	5 (3.3)
	PPROM	5 (3.3)
	Cholestasis of pregnancy	3 (2.0)
	Polyhydramnios	2 (1.3)
	IUGR	3 (2.0)
	Anencephaly	2 (1.3)
	Placenta previa	4 (2.6)
	More than one complication	3 (2.0)
Route of delivery	Cesarean section	108 (70.1)
	Cesarean section in singletons	48 (55.8)
Multiple pregnancy	Multiple gestation	83 (38.4)
Multiple gestation	Twins	69 (83.1)
	Triplets	13 (15.7)
	Quadruplets	1 (1.2)
Multiple fetal reduction	MFR	5 (2.9)

Data are presented as mean ± SD or n (%).

Received: 21/January/2019, Accepted: 3/August/2019
***Corresponding Address: Department of Obstetrics and Gynecology, American University of Beirut Medical Center, Beirut, Lebanon**
Email: gg02@aub.edu.lb

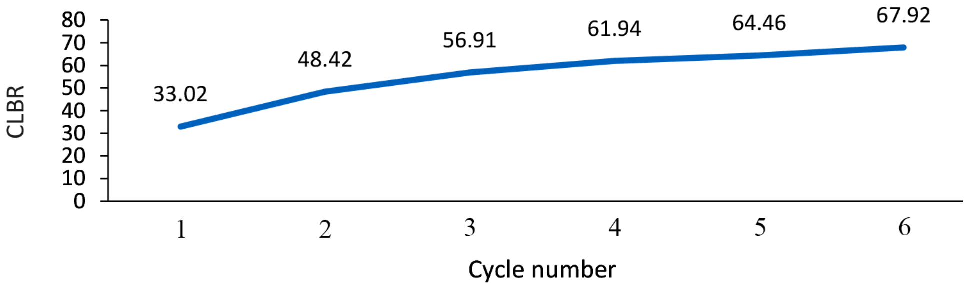

Fig.S1: Overall conservative cumulative live-birth rate (CLBR).

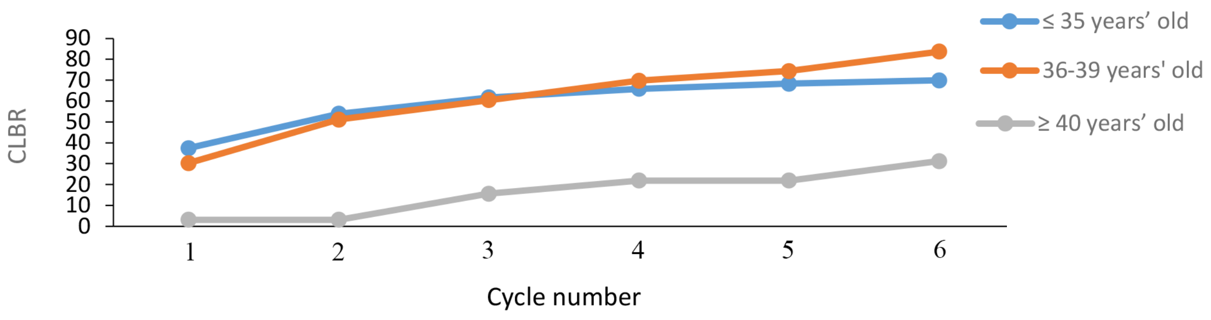

Fig.S2: Conservative cumulative live-birth rate (CLBR) according to the maternal age.

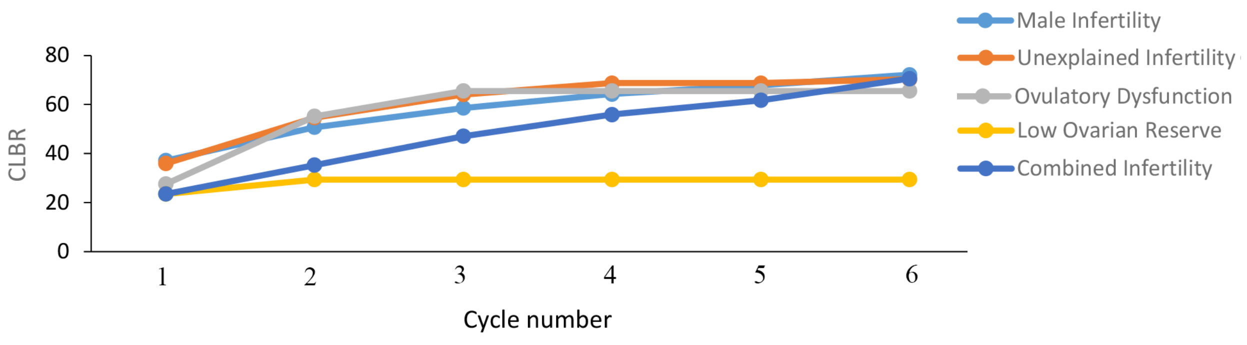

Fig.S3: Conservative cumulative live-birth rate (CLBR) according to the type of infertility.
